# Justification for a Nuclear Global Health Workforce: multidisciplinary analysis of risk, survivability & preparedness, with emphasis on the triage management of thermal burns

**DOI:** 10.1186/s13031-017-0116-y

**Published:** 2017-08-01

**Authors:** Frederick M. Burkle, Tom Potokar, James E. Gosney, Cham Dallas

**Affiliations:** 1 Harvard Humanitarian Initiative, Harvard University, Cambridge, MA USA; 2 Woodrow Wilson International Center for Scholars, Washington, DC USA; 3 Global Burn Injury Policy & Research, College of Human & Health Sciences, Consultant Burns Surgeon, Welsh Center for Burns & Plastic Surgery, Morriston Hospital, Swansea University, Swansea, Wales UK; 4 Disaster Medicine Rehabilitation, Norfolk, VA USA; 5 Institute for Disaster Management, Department of Health Policy and Management, College of Public Health, University of Georgia, Athens, GA USA; 6 Emergency Medicine, Department of Emergency Medicine, Medical College of Georgia, Georgia Regents University, Augusta, USA; 7 Harvard Humanitarian Initiative, c/o 452 Iana Street, Kailua, HI 96734 USA

**Keywords:** Global Health workforce, Nuclear war, Triage, Nuclear thermal burns, World Health Organization, Emergency medical teams

## Abstract

Major challenges and crises in global health will not be solved by health alone; requiring rather a multidisciplinary, evidence-based analytical approach to prevention, preparedness and response. One such potential crisis is the continued spread of nuclear weapons to more nations concurrent with the increased volatility of international relations that has significantly escalated the risk of a major nuclear weapon exchange. This study argues for the development of a multidisciplinary global health response agenda based on the reality of the current political analysis of nuclear risk, research evidence suggesting higher-than-expected survivability risk, and the potential for improved health outcomes based on medical advances. To date, the medical consequences of such an exchange are not credibly addressed by any nation at this time, despite recent advances. While no one country could mount such a response, an international body of responders organized in the same fashion as the current World Health Organization’s global health workforce initiative for large-scale natural and public health emergencies could enlist and train for just such an emergency. A Nuclear Global Health Workforce is described for addressing the unprecedented medical and public health needs to be expected in the event of a nuclear conflict or catastrophic accident. The example of addressing mass casualty nuclear thermal burns outlines the potential triage and clinical response management of survivors enabled by this global approach.

## Background

The humanitarian health professions learned many hard lessons from the Ebola epidemic in West Africa. Despite previous warnings from the 2003 SARS pandemic and the passage of the International Health Regulations Treaty in 2005 and updated in 2009 [1], poor Treaty compliance, lack of financial support, and leadership failures ultimately led to delays in recognition and response to the Ebola epidemic [2]. Post-Ebola appeals for a World Health Organization (WHO) led global health workforce resulted in 2014 of the development of country-supported international and national emergency medical teams (EMTs) for rapid and sustained response to sudden onset natural disasters (SoDs), public health emergencies of international concern (PHEICs) and conflict-related complex humanitarian emergencies (CHEs) [2]. In 2015, amid the increasing risk of a nuclear crisis with unmitigated and shared components of both CHEs and PHEICs, Burkle and Dallas argued for the inclusion within the WHO agenda of a nuclear global health workforce capacity and operational framework [3].

This study, based on current multidisciplinary analysis of geopolitical risk, scientific nuclear survivability research, and medical advances in both triage management and the acute and chronic care of nuclear thermal burns, as one clinical example, argues that life-saving opportunities are possible if rapidly deployed, justifying the development of a multidisciplinary nuclear global health workforce.

## Analysis of risk of nuclear war

As writers and watchdogs on nuclear risks and the health outcomes of nuclear war, we are worried. From a scientist’s standpoint, general ignorance of the medical and public health realities of nuclear tragedies has never been at such a dangerously high level. Since the 1980s and the Chernobyl disaster, at least 7 individual nuclear accidents including the Fukushima Daichi tragedy and Russian K-84 submarine accident have occurred. The breakup of the former Soviet Union led to less security over fissile materials and the “undetected smuggling of weapons-usable nuclear material or how much was diverted or stolen since” that may support global terrorism groups with the stated aim to utilize this technology [4]. The current “conventional wisdom is that a dirty bomb is far more likely than a conventional bomb” [5].

Rather than moving toward a “nuclear free world” the U.S. finds itself embarking on a $1 trillion nuclear weapons “modernization program” to counter increasing Russian provocations [6]. Every other nuclear power worldwide has either increased the size of their arsenal or modernized it [3]. While there has been an overall decline in the number of nuclear weapons worldwide, the steady expansion of nuclear stockpiles to more national states (including highly aggressive and arguably unstable ones), the continuous modernization of arsenals and a concurrent decline in regional stability, especially in the South Asian subcontinent, provides an increasingly dangerous flashpoint for nuclear war. The total number of nuclear weapons reached a peak in the 1980s, more countries now possess them. Risks of a nuclear tragedy are significantly building despite vigorous attempts at slowing proliferation.

India and Pakistan fought four wars over the four decades preceding their acquisition of nuclear weapons. The religious animosity is deep-seated, having lasted for over a thousand years. Today, while both nations have over a hundred nuclear weapons, all of them in the Hiroshima size range (15-20 kT yield), they are in the process of rapidly proliferating nuclear stockpiles. Pakistan is adding 20 weapons per year, the fastest numerical level of proliferation worldwide while India is poised to achieving thermonuclear weapons (>100 kT yield). Others regard Pakistan’s 2015 plan to deploy tactical short-range, low yield nuclear weapons (Less than 5 kT) as “problematic and risky” arguing that such weapons “lower the threshold for nuclear weapon use” [7]. This argument began in the new Century with the suggestion that these “low yield” weapons could be used in conventional conflicts, blurring the distinction between nuclear and conventional war [8]. Critics claim that “what smaller does is to make the weapon more thinkable” [9].

In addition to the intense rivalry with Pakistan, India also perceives a growing threat from dramatic increases in the military stature of China. This is further exacerbated by the highly publicized nuclear activities by Iran, which after IAEA-documented research efforts in nuclear weapon development, today Iran has the capability to develop a nuclear weapon [10]. Therefore, there exists a rapid and accelerating nuclear proliferation in the South Asian subcontinent where neither India nor Pakistan has signed the Comprehensive Nuclear Test Ban Treaty. Analysts worldwide are concerned for the stability of the Pakistani nuclear arsenal fuelled by growing tensions between its military and civilian leadership, tribal and radical Islamic warfare and the acknowledged presence of the followers of these groups in the Pakistan government [11]. The increasingly violent warfare between Sunni and Shiite Islamic forces in the Middle East and South Asia has led to anxiety over acquisition of nuclear weapons by wealthy and influential Sunni Saudi Arabia from Sunni Pakistan to counter the pursuit of nuclear weapons capability by Shiite Iran.

Beside the Cold War Northern hemisphere build up the steady increase in number and weapon size of South Asian nuclear arsenals in this crowded corner of the planet is otherwise unprecedented. Globally, “modernization” still refers to nuclear proliferation and it is now worldwide. An unnamed official from the Obama administration explained, “Why is it that we’re trying to prevent the Pakistani government from collapsing? Because we fundamentally believe that we cannot afford a country with 80-100 nuclear weapons becoming the Congo…there is a sense that other places in the world can go to hell, but not this one” [12].

Emerging conditions that mimic Cold War mindsets should make us increasingly wary. Russia’s military exercises and explicit threats against other countries and especially their vigorous nuclear modernization program reflects their conviction that strategic nuclear forces are indispensible for security and status as a great power [9]. Today, nuclear weapons and what they symbolically represent varies significantly from the regional power rivalries of the past. Most worrisome are that the possession of advanced nuclear weaponry, once steadfastly controlled by arguably rational regimes, has taken on more religious significance in countries where they are worshipped both as a national strength and as a right, or as a “currency of power to which many countries still aspire.” How such religious fervor might influence and justify their use has not been adequately researched, but should not limit the topic from being integrated into existing deliberations if that risk is to be mitigated [13].

It is also discouraging that American attitudes regarding nuclear weapons use, the so-called “nuclear taboo”, have changed since post-WWII when public opinion supported an aversion to the use of nuclear weapons. Current “instincts of the U.S. public, when facing our worst foes” have shifted with a “sizeable segment of the American public” feeling “an attraction to our most destructive weapons, not an aversion”. Previous “commitment to the immunity of civilians from deliberate attack in wartime, even with vast casualties, is shallow” [14].

## Survivability in nuclear war

Nuclear negotiators rarely mention the dire health outcomes, nor have the tragic health consequences ever been used as an agenda item or an incentive for nuclear non-proliferation negotiations. These actions suggest that denial, as a protective mechanism of most humans, is massive cross-culturally, even among seasoned health care providers when it comes to contemplating the possibility of a nuclear tragedy [15].

Recent scientific research assists in dispelling the thinking that started in the 1950s that nuclear conflicts would result in very few survivors. Today’s research supports that a nuclear war, even a catastrophic duel between Iran and Israel, would result in an extraordinary number of surviving radiation, trauma and thermal burn patients receiving almost no medical care or even pain relief [15–18].

One of the most pervasive perceptions left over from the Cold War is the consideration of the inevitability of high mortality and injury rates from nuclear war, in high proportion to the overall targeted population. This was due to the potential for large numbers (perhaps even thousands of weapons) of high yield nuclear devices (hundreds and in many cases thousands of kilotons each) being in a nuclear conflict between the superpowers. Known as Mutual Assured Destruction (MAD), it proved to be a powerful deterrent to the use of nuclear weapons during the decades after the Second World War. However, it also left a remarkably persistent perception that the use of nuclear weapons inevitably resulted in a high proportion of casualties to the population of the area attacked, even to the extent that medical and public health personnel, military planners, political thought leaders and policy makers, and other decision makers continue to operate under this assumption.

Today the U.S. Department of Homeland Security (DHS) has as its most likely scenario for a nuclear weapon detonation in the U.S. as a 10 kT nuclear weapon in an urban area [18]. It is also widely assumed that a single nuclear weapon detonation is the most likely nuclear event to be seen, with several of these relatively smaller weapons being used in a short period of time as the next most likely event. Put in a strictly quantitative framework, this means that it is incumbent on health and civil services to have sufficient emergency preparedness for a situation in which cumulative kiloton nuclear attack of from 10 to 50 kT occurs. This is strikingly different from the MAD structure of a total 5000,000 kT attack (1000 kT times 5000 weapons, still only a fraction of the total arsenal of our opponents in the Cold War). In this illustrative example, this is a 100,000-fold difference, or 5 orders of magnitude. Clearly, even with some considerable contraction of the breadth of difference, the remarkably disproportional impact of the health care impact in the Cold War and the current time must be acknowledged.

### Illustrative example of nuclear war survivability

Looking closely at the detailed health outcome of a 20 kT detonation in Washington, D.C., better brings into focus the reality of the current threat, as was done in a U.S. Senate Homeland Security Hearing given by co-author (CD) and at which the current Secretary of Defense was also a speaker [19]. In short, the high lethality and morbidity in the first mile or two around the blast area gives way to a narrow slice of area where actual radiation-induced casualties occur in a plume outside this inner zone, which actually covers only a small part of the city. In the first 750 m (12 psi) virtually all buildings will be destroyed by blast, mass fires are common and prompt radiation doses are fatal except in basements, resulting in very few survivors. Between 750 and 1250 m the peak overpressure decreases from 12 to 5 psi with walls blown out of buildings. Debris will be tens of feet thick in most downtown areas with ten story plus buildings. Roughly half of the population in this relatively limited area will be fatalities, mainly from collapsing buildings, with the other half injured. Most of those surviving will have been exposed to a fatal dose of prompt radiation, though death will occur first due to mass fires or third degree burns. Between 1250 m and 1750 m peak overpressures will fall from 5 psi to near 3 psi, and burn thresholds towards the edge of this zone will drop from third degree to second degree levels. At 1900 m or 3 psi, large numbers of trauma injury would ensue from walls blown out of steel framed buildings, severe residential damage and people caught in the open. By 2000 m burn risk will drop to first degree levels. At up to 3800 m or 1 psi people will be endangered with flying glass and debris from damaged structures and glass will break out to over 6 kms. The very high deposition rates of radioactive particles that occur in the first km from the detonation rapidly drops off in magnitude, along with the resulting radiation-induced casualties, with wind dispersion.

This description, however, accounts for only about 1 mile across of an urban area, with the rest of the sprawling urban landscape of U.S. cities as unaffected, with the exception of a relatively narrow plume of casualties induced by sufficient radioactive fallout that can continue to extend out for several miles. However, most people think of these effects extending for 10, 20 or more miles across an urban landscape in all directions. Using a clock-face analogy, the much-feared health effects from the radioactive plume for a 10- or 20-kT detonation with wind coming from the West would roughly approximate only a 2:00 to 4:00 region for several miles, with the rest of the clock-face outside the 1–2 mile radius around Ground Zero as completely unaffected in terms of direct health care consequences due immediately to the nuclear weapon detonation [20]. There would be extensive indirect and potentially preventable effects due to power grid loss, lack of access to food, water, sanitation, shelter, access to and availability of healthcare and medicines, increased crime, and the health issues related to internally displaced people, especially vulnerable populations [21].

The healthcare affected population in the Washington 20 kT scenario will entail a daunting task for response, but would actually involve only a fraction of the total population [22]. Nuclear war casualties can in this context be simplified to thermal burn, trauma, radiation casualties and combined injuries. Defining the mortality and surviving populations is inexact, but due to the large difference between the reasonably assumed mortality and the known unaffected areas, the error in these assumptions is not great. All thermal casualties would number about 40,000, with 25,000 of these as the more survivable first degree burns. The combined fallout and blast affected populations (dead and surviving injured) would number about 220,000. However, 50,000 of these are in the fallout zones where mortality is less than 10%. Another 60,000 of the blast casualties are in the 1-2 psi zone and most would be expected to survive. Therefore, in this rough approximation there are 260,000 combined thermal burn, trauma, and radiation affected population. Assuming 70% survival in the first degree burn (0.7 × 25,000 = 17,500) [17] and 1-2 psi zone trauma patients (0.7 × 60,000 = 42,000) and 90% survival in low fallout zones (0.9 × 50,000 = 45,000), means that subtracting these survivors from the total affected population leaves approximately 100,000 deaths. The 70% figure is an estimate of the survival rate for the combined injury of trauma from 1 to 2 psi earthquake survival rate together with the first degree burns. Most of the deaths would be due to the trauma, not the first degree burns, which are expected here to exacerbate the trauma injury severity [17]. It also leaves over 160,000 surviving injured likely requiring acute and chronic assistance. When one considers that the total metropolitan population of Washington, D.C., is over 6 million, this means that about 4% of the population of metropolitan Washington is likely to be affected by the most likely nuclear device to be used (10-20 kT), with 1.5% mortality and 2.5% injuries requiring assistance.

While a 1.5% mortality in a population is a devastating experience for any society, it is dramatically different from the common expectations one sees from the general population, or even educated medical circles, where MAD conditioning has left the impression of much higher mortality. It is incumbent on responsible medical and public health planners to acknowledge this discrepancy and put into practice (and support financially and with qualified personnel) the appropriate response to deal with what is a much more manageable, though still daunting, mass casualty medical response than previously acknowledged.

## Preparedness for nuclear global health workforce

The ICRC reminds us that for more than four decades during the Cold War preparedness drills were regularly conducted, shelters maintained and anti-nuclear protests took place. Two generations later, with most born after the end of the Cold War, the level of awareness of the risks to humanity are much less, especially in the U.S. but also worldwide [23]. Any one of a number of increasingly feasible regional conflicts with the growing list of nuclear powers, including increasingly aggressive ones more likely to use the weapons than in the past (India/Pakistan, Sunni/Shiite, Iran/Israel, Russia/NATO, North Korea) would expose an almost complete lack of preparedness of any of these nations to help the people who survive it. Nuclear tragedies result in the loss of basic command-and-control, mass surgical and trauma casualties suffering blast injuries, 1^st^, 2^nd^ and 3^rd^ degree thermal burns, radiation contamination, and multiple lacerations, orthopedic and crush injuries, severe combined effect injuries, and major psychosocial outcomes [3].

In addition to the fact that surviving physicians, nurses and emergency responders would be overwhelmed, most would lack the skills and the tools to treat the unprecedented number of thermal burn and radiation victims. Survivors in primarily “Injury Zones” would in a short time become “Death Zones” making the differences medically between the two zones almost meaningless [3]. This is obviously unacceptable to any observer, and demands a response from rational planners.

A nuclear event will result in an unprecedented mass casualty incident that produces large scale direct mortality and morbidity requiring the rapid deployment of mobile, self-contained, self-sufficient health care facilities and subsequently the more indirect health outcomes resulting from a destroyed essential public health infrastructure and the daily protections they afford society [24]. Hauer discusses the increasing likelihood of nuclear terrorism and how ill prepared the U.S. and other nations are to respond, asserting that “in most multi-casualty incidents, there are one or two triage points where victims are taken for assessment and routing to treatment. Following a nuclear detonation in a major city with a vast geographical footprint there could be need of 25 to 75 such triage points or more” [25]. Deployed emergency health facilities must have the capacity and capability to bring life-saving care as close to the frontline of the tragedy as is safely possible. In a series of 10 focus group discussions with ED physicians and nurses conducted in the USA in 2008, study participants consistently stated that neither ED ‘s nor hospital facilities were sufficiently prepared for a terrorist attack involving radioactive materials and expressed a need not only for more information but also strongly disagreed with some of the existing response guidelines [26]. Recommended requirements for centrally coordinated mobile and fixed initial triage and dose-monitoring facilities designed to identify, assess, transfer, decontaminate, and move casualties efficiently to survivor or palliative care facilities include: (1) Nuclear Triage Centers, (2) Nuclear Survival Centers, (3) Nuclear Palliative Care Centers, and (4) Health System Support Centers [3]. Recognizing the vast numbers of casualties that will occur, operationally all emergency medical teams would become resource scarce or constrained in a short period of time requiring unprecedented logistical resupply system that does not exist today. Existing International Atomic Energy Agency and WHO assets (field-level guidelines, nuclear triage and management plans, worksheets and technical monitoring standards for radiation overexposure) are available, however altered standards of care will be practiced at every level on a daily basis.

## Triage management of thermal burns enabled by a nuclear global health workforce

As an example of the potential impact of a Nuclear Global Health Workforce on constructing an effective medical response, the very difficult subset of nuclear war mass casualty thermal burn response is addressed. Over the decades since atomic bombs struck Hiroshima and Nagasaki one of the most common photographic reminders of these horrific events have been those of chronic keloid disfigurements resulting from thermal burns; reminders of both the suffering and survivability of nuclear war. Surviving medical professionals would be expected to be limited to treating lacerations and fractures, leaving those with severe thermal burns to die slowly of infection. It should be recognized that radiation burns also occur after a nuclear detonation, where a skin burn occurs due to ionizing radiation and not thermal energy, and that the overwhelming majority of these “burn” victims do not survive. In contrast, the thermal burn victims include a large number of surviving patients that can greatly benefit from appropriate medical intervention. Actually this is a fairly common mistake made in nuclear detonation discussions, where radiation and thermal burn statements are made interchangeably. From a triage point of view it is greatly simplified by the fact that the very large radiation dose required to generate a radiation burn insures that the patient will not likely survive, while fairly low exposures to heat energy will create a surviving thermal burn patient. To avoid the confusion which sometimes occurs in “burn” discussions related to a nuclear detonation, in all future burn discussion here, thermal burns are being referred to, and not radiation burns. Jeng and colleagues emphasize that triage decisions need to be made carefully, allowing the focus of limited personnel and equipment on those most likely to survive [27].

With current triage approaches, it is highly unlikely that the very large number of thermal burn victims will receive any meaningful medical treatment suggesting that a lower-tech approach could save many lives and involve training armies of non-specialists in surgical debridement and the administration of burn medicines [3]. Burn treatment as a high-tech endeavour would only be pursued in well-equipped hospital units where each patient gets constant attention from several specialists [3].

Yet, society recognizes legal, ethical and moral expectations and obligations that during crises triage plans exist to treat as many victims as possible who have an opportunity for survival. When triage is performed in accordance with accepted medical practice, triage is both sanctioned and recognized by law in most countries. Because unrealistic triage results in unacceptable deaths rates among those who should survive, triage plans must be well thought out and designed. In fact, medical providers are held legally accountable for the triage process where there is rule of law, but the process itself cannot ensure either treatment or survival [28]. In 1986, Leaning described in detail the unique challenges health care providers face in the triage and treatment of casualties from nuclear war. She stressed that “reference to precedent may admit sources of serious error, since in terms of scale of effects, this disaster would depart fundamentally from anything the world has yet experienced” further observing that whereas radiation injury must receive substantial attention, a synergism exists between burn and blast and the triage category of “expectant” may extend over a wide range of injury, including many who might in other, less-stressed circumstances be assigned greater chances of survival” [29]. Kumar and Jagetia contend that it will be very difficult to save victims with a TBSA of more than 30% and exposure of more than 4Gy of ionising radiation. Most available medical resources should be diverted to treating less severe and moderate combined injuries [30].

Coleman and colleagues provide realistic and thoughtful accounts of the current capacity, capability and future requirements necessary to sort, assess, treat, triage, monitor, recover and rehabilitate casualties and the public health protections after a nuclear attack [31–33]. They emphasize the criticality of triage in resource poor settings involving extensive preplanning, stating in 2016 that “an understanding of the requirements for biodosimetry, triage and treatment decisions, and massive public health and medical response informs and modifies the over-all large-scale operational response” [31]. While it is unfortunate that in most of the existing healthcare communities these skill sets needed in nuclear war medical response are simply lacking, it is entirely feasible that they could be gained by the appropriate training protocols and especially in the kind of international cooperative venues such as mass casualty exercises which require extensive planning and preliminary training. Therefore, in order to address these demanding resource needs it would be of high utility to mobilize the existing civilian and military healthcare personnel in not only the immediate affected nation but also in cooperating additional nations in a coordinated manner. A critical feature toward this end would be to incorporate retired healthcare personnel and the mature voluntary aid societies in well organized training paradigms and especially mass casualty management exercises with the existing healthcare sector. This would enable the twin needs of greatly expanding the number of qualified healthcare participants that are needed, and adding the critical missing training skill sets such as dosimetry and mass casualty triage and treatment to this expanded population. As it is established that a single nuclear event will exceed the capacity of any single nation to respond, this also enables the bringing together of the necessary volume as well as appropriate quality of healthcare resources in a viable organized fashion. We suggest that to effectively operationalize such an endeavor requires the necessity of a nuclear global health workforce organized, trained and supervised by the larger global network of governmental and non-governmental resources.

## Advances in thermal burn management to incorporate into a nuclear global health workforce

The management of burns has improved dramatically over the last 50 years, to the extent that in a fully resourced modern burns centre in a high income country burns of up to 80% TBSA can regularly survive. The developments that have led to these improvements have been stepwise and summative, but include early fluid resuscitation, early excision and grafting, management of sepsis, management of inhalation injury, nutritional support, use of cadaveric allograft and artificial dermal templates. In addition the creation of well trained dedicated multidisciplinary burn teams and advances in rehabilitation, scar management and psychosocial support has meant that not only has survival improved, but also quality of life post burn injury.

However, it is highly unlikely given the current circumstances that these gold standard modern burn management practices would be available after a nuclear disaster. In addition there are already enormous discrepancies between what is available in a high income versus resource poor country. Access to burn services and outcomes from burn injury in most of the world is poor with lack of trained staff, negligible pre hospital care, transport difficulties and insufficient materials – in other words not dissimilar to the situation after a nuclear incident.

The response to a medically overwhelming number of burn casualties after release of a nuclear device cannot therefore be limited to existing burns services which if not destroyed in the initial explosion would be totally overwhelmed within minutes. Therefore there is need for a complete paradigm shift in thinking and preparedness preparation. As noted previously, the most likely nuclear weapons to be used in the near future are the relatively smaller (Hiroshima/Nagasaki-sized) devices, which not only produce much fewer casualties than thermonuclear devices, but also a much smaller proportion of thermal burns as part of the total injuries of these larger nuclear devices.

Triage is clearly critical and has to be realistic, based on survivability and available resources (Table 1). The most impactful approach is likely to be simple, early management of the large number of relatively superficial flash burns, up to 30% TBSA and associated with minimal or no contamination or other injuries (unless very minor). Taking the example of a 20 Kt explosion in a city such as Washington, DC, the predicted number of people (of all ages) with superficial burns would be 25,000 – most likely dazed, confused, psychologically disturbed and in an environment where almost all of the infrastructure has been destroyed. Clearly these numbers are way beyond what can be managed by a single emergency medical team, but this cohort of patients are potentially amenable to either ‘self treatment’ or ‘buddy treatment’ and therefore one of the roles of the EMT would be to rapidly distribute essential treatment packages along with very simple instructions. In this scenario early oral resuscitation is likely to be more practical and achievable than intravenous resuscitation, although if there has been radiation exposure vomiting may be a problem [27, 34]. Analgesia should be available and instructions given on cleaning wounds and applying an antimicrobial ointment with closed dressings. There are numerous modern silver impregnated dressings that are more suitable for superficial partial thickness injuries that once applied can be left for 5–7 days which may well be more appropriate but are significantly more costly. Part of preparedness development would be identifying the exact contents of such ‘individual emergency burn packs’. The other large group of potentially survivable burns would be those with either larger surface area superficial burns or deeper burns most likely up to a maximum of 40% TBSA. In a low resource environment deep burns beyond this rarely survive even in a moderately well equipped and staffed burns services. The second role of a burn specific EMT would then be to deal with these patients. It is feasible within a field hospital setting to undertake excision and grafting of these patients, and early excision would be preferable as this not only improves outcomes and mortality in general, but also reduces infective complications [35]. There have also been advances in human fibroblast-derived temporary skin substitutes, with potential applications in mass casualty burn treatment as has been demonstrated after fire-fighter thermal burn treatment [36, 37]. The use of these skin substitutes might have feasibility in the treatment of the potentially large number of burn victims in a nuclear scenario, as their use may feasibly delay the inflammatory response in treated patients long enough to enable the mass casualty transport and triage delays until more conventional and effective burn treatments can occur. If there has been concomitant low level irradiation then the impact on the immune system will be delayed by 4–6 days, so ideally patients should be excised and grafted before this stage. With the potential of several thousand patients requiring early excision and grafting, it is clear that multiple EMT’s would be needed to perform this; however, appropriate triage and rapid simple surgery could potentially save a significant number of these patients. Combined thermal and irradiation injuries have a worse prognosis, whilst pure radiation burns are not like thermal burns in that the radiation kills the basal layer of dividing cells and therefore desquamation is not seen for several days and this type of injury usually result in superficial burns only [38].

**Table 1:** Potential roles of EMT in aftermath of thermonuclear event

Patient Category	Immediate Management	Where	Role of EMT
Superficial burns up to 30% TBSA	Oral resuscitation if over 15%, analgesia, dressings, availability of antibiotics	Ambulatory	Distribution and instruction in use of ‘individual emergency burn pack’
Superficial burns over 30% TBSA and deep burns up to 40% TBSA	Oral resuscitation if over 15%, analgesia, dressings, availability of antibiotics PLUS early excision and grafting of deep burns	Field Hospital	Manage resuscitation, clean and dress wounds, early excision and grafting. Rapid training of temporary burn care teams to assist with dressings, positioning, mobilising, hygiene, feeding, etc.
Deep burns over 40% TBSA	IV fluid resuscitation, analgesia dressings and transfer if possible	Transfer out of area to functioning specialist burns service	Confirm triage to ensure appropriate use of very limited transport and specialist services

*TBSA* total body surface area

In the event of only a fraction of the thermally injured surviving (say 10%) this would still equate to between 2500 and 4000 patients in the scenario described above. These patients will then need ongoing care and rehabilitation. Burns that have healed within 2 weeks are unlikely to need ongoing management beyond application of simple moisturisers and advice on skin care and protection. However, deeper burns and those taking longer than 2 weeks will need early rehabilitation, ideally starting with appropriate positioning and/or splinting, mobilisation and nutritional support. Again, it is clear that in the acute stages access to standard nursing and therapy care will not be possible and therefore alternative approaches will be required. EMT’s with appropriate training could create ‘temporary burn care teams’ from surviving casualties (and indeed patients with only minor burns) and provide simple instructions and demonstrations of key points with respect to nursing care and acute rehabilitation (focussing on preventing problems further down the line).

Current burn mass casualty plans that have been developed do not consider the same level of magnitude of casualties that would be the inevitable result of a nuclear attack. Post 9/11 ‘bypass’ strategies have been replaced with the concept of ‘absorption’ strategies and developing surge capacity. However, these still focus on ‘traditional’ concepts such as moving patients with serious burn injuries to a burns centre and keeping those less severe in outlying hospitals until beds become available in a burns center [39].

In the USA there are only 128 burn centers (with a total burn-bed capacity of 1835) and only 43 burn centers are verified by the American Burn Association and the American College of Surgeons through a rigorous review program designed to determine if the burn center’s resources are sufficient to provide optimal care for burn patients [19]. In the 20 kT example given above there would be a possible 15,000 patients with burns deeper than superficial and thus potentially requiring surgery and if even 10% of these survived, it would still overwhelm the existing burn services, thus a very different model of managing these cases to maximise potential survival must be seriously considered.

It is in the survivability consideration of the relatively smaller nuclear weapons mentioned earlier that a curious and unexpected incentive emerges for vigorously enacting a nuclear response training and exercise paradigm for mass casualty thermal burn and other protocols. As nuclear detonation events become more likely, it is ironically fortunate that it is single or small numbers of the smaller, Hiroshima-sized weapons that are reasonably expected to occur at least in the initial nuclear events over the next decade or so. The number of burn patients generated by these scenarios, while still catastrophic, will enable a credible response by a properly prepared global health workforce. An encouraging example toward this end is the WHO EMT initiative, which operates on a regional basis through WHO regional organizations, and enables the mobilization of other resources outside that region, as would certainly be necessary in a nuclear event. As these resources are trained globally in a common network of terminology and procedure, they have a common basis for cooperation and combination of forces in a mass casualty crisis such as a nuclear event. This approach should therefore be able to significantly help enable the medical and public health community to lead their respective communities into this future, and make it much less daunting than is currently perceived. Therefore, creating a nuclear global health workforce would be a critical first step in help regional healthcare response entities in developing realistic new strategies to deal with the inevitable aftermath of a catastrophic casualty scenario after a thermonuclear event [3].

## Conclusions

The risk of a nuclear event, either by accident or war, has increased. This comes as evidence of increased survivability from the most likely initial nuclear weapon events has emerged. Multidisciplinary prevention, preparedness and response, however, remain a major deficit. Using the example of triage and management of one of the more dreadful of its consequences, thermal burns, the authors strongly recommend the development of a WHO sponsored nuclear global health workforce.
